# Complete Mitochondrial Genome of *Trichuris* *trichiura* from *Macaca sylvanus* and *Papio papio*

**DOI:** 10.3390/life11020126

**Published:** 2021-02-06

**Authors:** Julia Rivero, Rocío Callejón, Cristina Cutillas

**Affiliations:** Department of Microbiology and Parasitology, Faculty of Pharmacy, University of Seville, Calle San Fernando, 4, 41004 Sevilla, Spain; jrfernandez@us.es (J.R.); callejon@us.es (R.C.)

**Keywords:** complete mitochondrial genome, *Trichuris trichiura*, phylogeny, whipworms

## Abstract

Trichuriasis is among the most prevalent worldwide parasitism caused by helminths. For many years, *Trichuris* spp. have been described with a relatively narrow range of both morphological and biometrical features. The use of the complete mitochondrial genome (mitogenome) is an alternative and powerful molecular method for inferring phylogenies. Here, we present an overview of the contributions of mitogenome for *Trichuris* spp. from human and non-human primates. In addition, we carry out structural and phylogenetic comparative analyses with genomes of *Trichuris* species available in public datasets. The complete mt genomes of *Trichuris trichiura* and *Trichuris* sp. from *Macaca sylvanus* and *T. trichiura* from *Papio papio* are 14,091 bp, 14,047 bp and 14,089 bp in length, respectively. The three mt genomes are circular and consist of 37 genes—13 PCGs (*cox*1–3, *nad*1–6, *nad*4L, *atp*6, *atp*8 and *co*b), 22 transfer RNA genes (tRNAs), and two rRNAs (*rrn*L and *rrn*S). The molecular evidence presented here supports the hypothesis that *T. trichiura* de *M. sylvanus* (TMF31) and *T. trichiura* de *P. papio* (TPM1) were similar but genetically different with respect to *Trichuris* sp. from macaques (TMM5). The phylogenetic study also supported the evolution of the different *Trichuris* species. In conclusion, we suggest the existence of two cryptic species parasitizing *M. sylvanus*.

## 1. Introduction

Soil-transmitted helminths (STHs) (*Ascaris lumbricoides* (Ascariasis), *Trichuris trichiura* (Trichuriasis)) and hookworms (Ancylostomiasis/Necatoriasis) are widely distributed in tropical and subtropical areas, with the greatest numbers occurring in sub-Saharan Africa, the Americas, China, and East Asia [1]. In areas with favorable climatic and environmental conditions, poor access to potable water supply, sanitation, and hygiene resources, the transmission of these parasites is higher [2]. Recent data show that about 1.5 billion people are estimated to be affected by STHs worldwide [1].

Although Trichuriasis is a seriously neglected disease, fortunately, in recent years hereditary studies are increasing as well as related methodologies that provide new opportunities for the discovery of novel intervention strategies, with major implications for improving animal and human health and welfare globally [3]. In addition, the implications of genomic studies could also be very relevant in relation to the search for new treatments for immunopathological diseases in humans [4,5,6,7,8,9]. For example, it has been reported that infections of human patients suffering from immunological disorders (such as Crohn’s disease) suppress clinical symptoms significantly with pig-*Trichuris* eggs [6,10,11,12].

The nematode mitogenome (complete mitochondrial genome) has several practical strengths as a phylogenetic marker, and has yielded well-supported results for clades, which were not well resolved using other approaches [13]. The whole genome and transcriptome studies have contributed to our understanding of deep node nematode phylogeny but have been limited by low taxon sampling (which is also biased toward some parasitic groups) and by the technical challenges inherent in obtaining a large quantity of genetic information from a single nematode with a small body size. In such cases, the use of mitogenome sequences is one alternative that has been widely applied to many nematode branches where relationships were unclear [13].

Nematode mitogenomes are like those of other animals in many respects, but have a few unusual features including high variation in conservation of gene order across major branches and the occasional presence of multiple chromosomes [13]. The nematode mitogenome is usually a single, circular molecule ranging in size from 12 to 22 kb and containing 36 (sometimes 37) genes—12 (or 13) protein coding genes (PCGs) (*cox*1–*co*x3, *cyt*b, *nad*1-*nad*6, *nad*4L, *atp*6, and rarely *atp*8), two ribosomal RNA (rRNA) (*rrn*L and *rrn*S), and 22 transfer RNA genes (tRNAs). The *atp*8 gene, which is found in most other metazoan mitogenomes (except the parasitic Platyhelminthes clade Neodermata) [14], is usually absent in nematode mitogenomes, although it does appear in the order Trichinellida (*Trichinella* spp. and *Trichuris* spp.) [13,15,16,17,18,19,20]. Furthermore, within class Enoplea, members of order Trichinellida (*Trichinell*a spp., *Trichuris* spp.) show a substantial gene rearrangement even among closely related species, while members of Chromadorea show far less rearrangement in theirs mitogenomes [21].

Kern et al. [13] concluded that the mitochondrial genome is a useful tool for nematode phylogenetic because the diversity within nematode mitogenome architecture, its variable rate of gene rearrangement, and the representation of nearly every kind of lifestyle and habitat ecology within nematodes make this phylum an exciting area for addressing questions about mitogenome evolution.

Currently, the complete mt genome of several species of whipworms has been sequenced—*Trichuris trichiura* from humans and baboons [3,19]; *Trichuris* sp. from the Endangered François’ Leaf-Monkey [17]; *Trichuris rhinopiptheroxella* from the endangered golden snub-nosed monkey [20]; *Trichuris suis* from pig [3,19]; *Trichuris ovis* from antelope [16]; *Trichuris discolor* from wild yak [16]; *Trichuris skrjabini* from sheep [22]; and *Trichuris muris* (LC050561, unpublished). This fact, and considering the hypothesis that *Trichuris* infecting primates represent a complex of cryptic species with some species capable of infecting both humans and non-human primates (NHPs), reflects the need to delve into the mt genomes of *Trichuris* spp. of NHPs compared to *T. trichiura* from humans.

Thus, the aim of the present study was to sequence the complete mt genome of *Trichuris trichiura* isolated from different NHPs. In addition, we carry out structural and phylogenetic comparative analyses with genomes of species of genus *Trichuris* available in public datasets.

## 2. Materials and Methods

### 2.1. Ethics Statement

This study does not require approval by an ethics committee. *Macaca sylvanus*, from which *Trichuris* specimens were collected from their caeca post-mortem. *Trichuris* specimens, recovered from the feces of one *Papio papio* after anthelmintic treatment, were handled and housed in a zoo in strict accordance with good animal practices.

### 2.2. Parasites, DNA Extraction and Genotyping of Worms

Specimen *Trichuris* worms were recollected from a Guinea baboon (*P. papio*) at Parque de la Naturaleza de Cabárceno (Cantabria, Spain), from a stool sample after anthelmintic treatment, and a Barbary macaque (*M. sylvanus*) in Castellar Zoo (Cádiz, Spain), from its caeca post-mortem. Adult *Trichuris* were recovered and washed in physiological saline, identified morphologically, and then, stored at −20 °C until use. Total genomic DNA was isolated from three individual worms according to the manufacturer’s protocol the DNeasy Blood and Tissue Kit (Qiagen, Hilden, Düsseldorf, Germany),used to extract the genomic DNA. Quality of extractions was assessed using 0.8% agarose gel electrophoresis infused with SYBR^®^ Safe DNA gel stain (Thermo Fisher Scientific, Waltham, MA, USA)

### 2.3. Mitochondrial Genome Amplification and Sequencing

For long-range PCR amplification and next generation sequencing (NGS), different primate derived *Trichuris* specimens were chosen. One Guinea baboon worm (TPM1) and two Barbary macaque worms (TMF31 and TMM5) were chosen based on their distinct haplotypes identified in previous studies by sequencing the partial gene of mtDNA (*cox*1, *co*b and *rrn*L) and rDNA (ITS1 and ITS2) [23]. To obtain the complete mt genome, firstly, we used the primers designed by Hawash et al. [19] to amplify the *Trichuris* sp. from baboon (TTB1) and *T. trichiura* from humans from Uganda (TTHUG) genomes in two overlapping fragments (~8 and ~6 kbp), Nevertheless, we only could amplify the second fragment (~6 kbp), from *rrn*L to *nad*1. Then, new primers (MS1F and MS1R) were designed to amplify the other fragment using Primer3 from *nad*1 to *rrn*L (http://bioinfo.ut.ee/primer3-0.4.0/ (accessed on 1 February 2021)). PCR mix, PCR conditions and PCR primers are summarized in the Supplementary materials (Table S1). The PCR products were checked on SYBR^®^ Safe stained 0.8% Tris-Borate-EDTA (TBE) agarose gels and bands were eluted and purified from the agarose gel using the QWizard SV Gel and PCR Clean Up System Kit (Promega, Madison, WI, USA). Once purified, the samples were concentrated and measured using a NanoDrop 2000 spectrophotometer (Thermo Fisher Scientific, Waltham, MA, USA). Stab Vida (Lisbon, Portugal) sequenced the mt genomes. The PCR products were used for library construction using Illumina Nextera XT library preparation kit (San Diego, CA, USA) and the generated DNA fragments were sequenced in the Illumina MiSeq platform (San Diego, CA, USA), using 300 bp paired-end sequencing reads.

### 2.4. Assembly, Annotation, and Genome Sequence Analyses

Sequences were assembled and analyzed using MacVector package v17.5.4 (Oxford Molecular Group, Waterbeach, Cambridge, UK). The identity of the sequences was made using BLAST by comparison with other sequences available in GenBank database. Genome annotation was performed using the pipeline MITOS web server (http://mitos.bioinf.uni-leipzig.de/index.py (accessed on 1 February 2021)) [24] using the mitochondrial invertebrate genetic code, and MacVector and protein-encoding genes of *T. trichiura*. The majority of the tRNA genes were identified using the ARWEN tool (http://130.235.244.92/ARWEN/ (accessed on 1 February 2021)) to get rRNA [25] and tRNAScan-SE web server (http://trna.ucsc.edu/tRNAscan-SE/ (accessed on 1 February 2021)) [26], but the remaining tRNA genes were recognized manually by comparison.

The genomes were compared with *T. trichiura* and *Trichuris* sp. Sequences—*T. trichiura* from humans (unknown geographical location) (AP017704), *T. trichiura* from humans in China (NC_017750), *T. trichiura* from humans in China (GU385218), *T. trichiura* from humans in Uganda (KT449826), *T. trichiura* from *P. anubis* in USA (KT449825), *T. trichiura* from *P. hamadryas* in Denmark (KT449824) and *Trichuris* sp. from *T. francoisi* in China (KC461179). All protein-coding genes (PCGs) and ribosomal RNA genes (rRNAs) sequences were individually extracted, and a nucleotide data set was generated by concatenated sequences (all PCGs and rRNAs genes). This data set was used to estimate genetic distances using MEGA X v.10.1.8 (Penn State, PA, USA) [27]. The genomes of *Trichuris* from humans and NHP were used to calculate the < Nucleotide diversity (π) using a sliding window of 100 bp with 25 bp steps implemented in DnaSP v.6 [28].

### 2.5. Phylogenetic Analysis

For the phylogenetic analyses, two different data sets were generated. The first included nucleotides sequences, with the 13 PCGs (*cox*1–3, *nad*1–6, *nad*4L, *atp*6, *atp*8 and *co*b) and the two rRNAs (*rrn*L and *rrn*S). The data set was aligned using MEGA X v.10.1.8 [27] and concatenated for *Trichuris* sp. and *T. trichiura* species from baboons and humans. We used as an outgroup *Trichinella pseudospiralis* (Table 1).

Another data set was generated using amino acid sequences inferred from the 12 PCGs. The sequences were aligned using MEGA X v.10.1.8 [27] and then concatenated excluding *atp*8 (because it is not present in the mitochondrial genome of all the species of nematodes except in *Trichinella* and *Trichuris* species). The data set, using the sequences previously used and all the sequences available of the complete mt genome of *Trichuris* spp. and with those of 9 other enoplid nematodes, using *Brugia malayi* and *Ascaris suum* as the outgroups, was generated (Table 2). The ambiguous regions of the alignment were excluded using Gblocks Server v.0.91b (http://phylogeny.lirmm.fr/phylo_cgi/one_task.cgi?task_type=gblocks (accessed on 1 February 2021)) with the default settings being used to select the option of less strict conservation of flanking positions [29,30].

For phylogenetic re-constructions we used three methods—Maximum Likelihood (ML), Maximum Parsimony (MP) and Bayesian Inferences (BI). The ML tree was generated using PHYML package [31,32], and for the MP tree we used MEGA X v.10.1.8 [27] and for BI we used MrBayes v.3.2.6. [33]. To resolve the best-fit substitution model for the nucleotide data set we employed jModelTest [34] and ProtTest 3.4 for the amino acid data set. Models of evolution were defined according to the Akaike Information Criterion [35,36]. For the nucleotide data set, GTR + I + G model, with rate variation along the length of the alignment (+ G) and allowing for a proportion of invariant sites (+ I) was selected, and for the amino acid data set, the MtArt + I + G + F model, with residue frequencies estimated from the data (+ F) was chosen. Support for the topology was examined using bootstrapping (heuristic option) [37] over 1000 replications to assess the relative reliability of clades. The commands used in MrBayes for BI were *nst* = mixed. The standard deviation of split frequencies was used to determine whether the number of generations completed was sufficient; the chain was sampled every 500 generations and each dataset was run for 10 million generations. Trees from the first million generations were discarded based on an assessment of convergence. Burn-in was determined empirically by examination of the log likelihood values of the chains. The Bayesian posterior probabilities (BPPs) comprise the percentage converted.

## 3. Results

### 3.1. Annotation and Features of Mitochondrial Genomes

**Figure 1:**
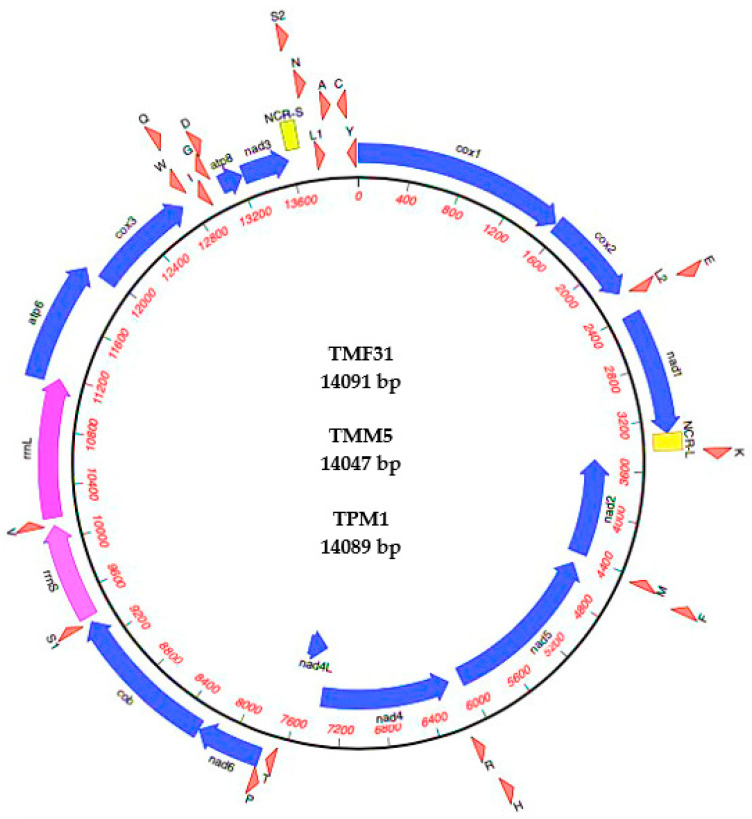
Mitochondrial genome structure of *Trichuris trichiura* from Barbary macaque (TMF31) and from Guinea baboon (TPM1), and *Trichuris* sp. from Barbary macaque (TMM5). Genes were represented to standard nomenclature, but tRNAs were represented using one-letter amino acid codes, with numbers differentiating each of the two leucine- and serine-specifying tRNAs. NCR-L refers to large non-coding region and NCR-S to small non-coding region.

The complete mtDNA sequences of the primate worms TMF31, TMM5 and TPM1 were 14,091, 14,047 and 14,089 bp in length, respectively (GenBank accession nos. MW448470-2) (Figure 1). The mt genomes contained 37 genes—13 PCGs (*cox*1–3, *nad*1–6, *nad*4L, *atp*6, *atp*8 and *cob*), 22 transfer RNA genes (tRNAs), and two rRNAs (*rrn*L and *rrn*S) (Table 3). All genes are transcribed from the heavy strand, except four PCGs (*nad*2, *nad*4, *nad*4L and *nad*5) and 10 tRNA (tRNA-Met, tRNA-Phe, tRNA-His, tRNA-Arg, tRNA-Pro, tRNA-Trp, tRNA-Ile, tRNA-Gly, tRNA-Cys, and tRNA-Tyr) that are transcribed from the light strand.

The mt genomes of *Trichuris* sp. contain an AT-rich region consisting of two non-coding regions (NCRs), including a long non-coding region (NCR-L) and short non-coding region (NCR-S). The nucleotide composition (%) is summarized in Table S3. The content of A + T is 69.4%, 68% and 69.3% for TMH31, TMM5 and TPM1, respectively.

Between TMF31 and TPM1 genomes there were slight differences. The sequences were identical in terms of all initiation and termination codons and gene lengths, except for tRNA-ser (S2), which presented one nucleotide more in TPM1. In addition, between TMF31 and TMM5, 22 genes of 37 were different in length.

The start/stop codons for some PCGs differed between TMM5 and the genomes previously cited (TMF31 and TPM1), which were similar. Codon usage analyses of mt genomes showed that three start and seven termination codons were different (Table 3). For instance, the starting codon for TMF31 and TPM1 is ATA for the *nad*1 gene, while it reads ATG in the TMM5 genome; for the *nad*4 gene, the starting codon is ATG in TMF31 and TPM1, while being ATA in TMM5, and in the *atp*8 gene, ATT is the start codon in TMF31 and TPM1, while it reads ATA in TMM5. In the *cox*1 gene, TAA is the termination codon in TMF31 and TPM1, whereas it is TAG in TMM5; in *cox*2 gene, TAG is the stop codon in TMF31 and TPM1, while being TAA in TMM5; in *nad*1, TAG is the stop codon in TMF31 and TPM1, but is TAA in TMM5; in *nad*2 gene, TAA is the stop codon in TMF31 and TPM1, and is TAG in TMM5; the stop codon in the *nad*5 gene is TAG for TMF31 and TPM1 and TAA for TMM5; in the *nad*4L and *nad*6 genes, TAA is the stop codon in TMF31 and TPM1 while it reads TAG in TMM5, and in *cox*3 gene TAA is the stop codon for TMF31 and TPM1 and TAG for TMM5. There are overlaps between *rrn*L and *atp*6 in TMF31, between *rrn*L, *atp*6 and *cox*3 in TMM5, and between *rrn*L and *atp*6 in TPM1.

### 3.2. Comparative Sequence Analyses

Genetic distances between worms for individual PCGs and rRNAs genes are found in Table S2. The genetic distances between the mt genomes of *Trichuris* spp. in primates and humans are given in Table 4. The gene with highest genetic variation was the *atp*8 gene, and the most conserved was the *rrn*S gene, however between all PCGs, *cox*1 was the most conserved gene. Among the mt genomes obtained in this study, nucleotide and amino acid differences between TMF31 and TPM1 were 0.25% and 0.41%, and between TMF31 and TMM5 they were 18.7% and 14.5%, respectively. Within all sequences of *Trichuris* spp. studied, the mt genome of *T. francoisi* was the most variable (with a nucleotide difference of 27.1–28.6% and an amino acid difference of 26.8–28.2%) relative to the other worms from baboons and humans.

**Figure 2:**
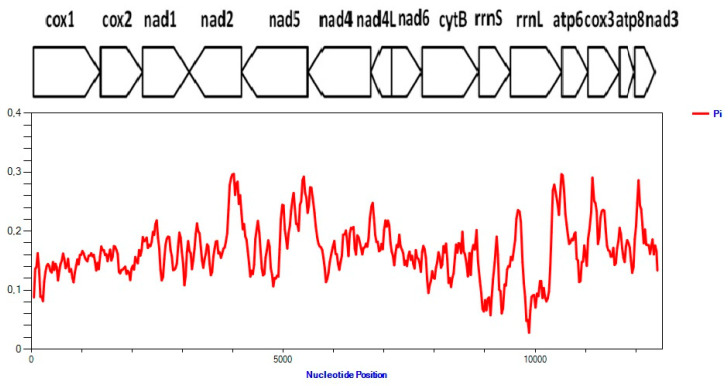
Nucleotide diversity (π) for all protein-coding genes (PCGs) and ribosomal rRNA (*rrn*S and *rrn*L) measured using a sliding window of 100 bp with 25 bp steps. The aligned dataset for *Trichuris* in primates (baboons, humans and Francois’ leaf monkey).

Among the *Trichuris* genome dataset, the nucleotide diversity was analyzed using the sliding window approach for the 13 PCGs and the two rRNA genes. The number of polymorphic sites was 5108 and the nucleotide diversity was 0.166 (Figure 2). The genes with lowest nucleotide diversity were rRNAs (*rrn*L and *rrn*S) and *cox*1.

### 3.3. Phylogenetic Analyses

**Figure 3:**
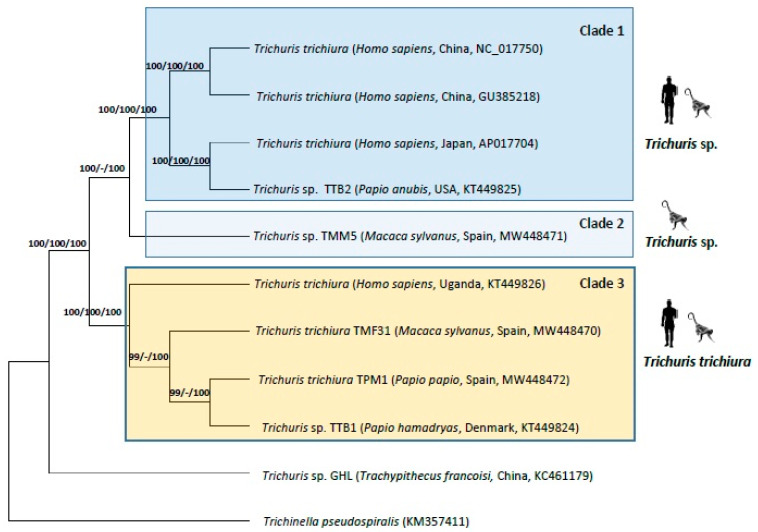
Phylogenetic tree based on concatenated nucleotide sequences of 13 PCGs and two rRNA of *Trichuris* from baboons, humans and françois’ leaf monkey using *Trichinella pseudospiralis* as an outgroup, inferred using Bayesian Inference (BI). Maximum Likelihood (ML) bootstrap values of clades are listed first, followed by Maximum Parsimony (MP) and by Bayesian Posterior Probabilities (BPP), for clade frequencies exceeding 60%.

The phylogenetics analyses of nucleotide sequence datasets with partial genome (the 13 PCGs (*cox*1–3, *nad*1–6, *nad*4L, *atp*6, *atp*8 and *cyt*b) and the two rRNAs (*rrn*L and *rrn*S)) and with mt genome complete datasets reflecting similar tree topologies by the three methods studied (ML, MP and BI) (Figure 3). Within *Trichuris* populations from humans and NHPs, *Trichuris* sp. from *T. francoisi* appeared separated of the other sequences. Within this last group, there are three main clades. The first clade (clade 1) is composed of sequences of *Trichuris* sp. from *H. sapiens* and *P. anubis* from Asia, Japan and USA, the clade 2 is composed of a sequence of *Trichuris* sp. from *M. sylvanus*, and the clade 3 corresponded with *T. trichiura* from humans and NHPs (*M. sylvanus*, *P. papio* and *P. hamadryas)* from Europe and Africa.

**Figure 4:**
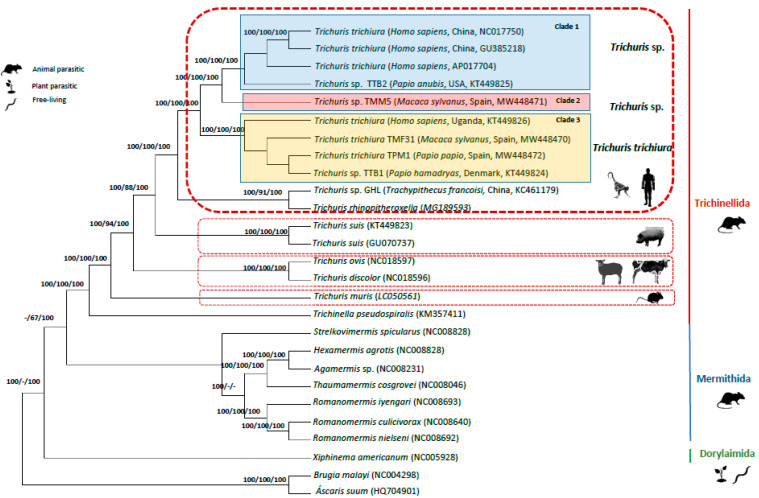
Phylogenetic tree among enoplid nematodes based on concatenated amino acid sequences of 12 PCGs (except for *atp*8 gene) by Bayesian Inference (BI) using *Brugia malayi* and *Ascaris suum* as the outgroups. Maximum Likelihood (ML) bootstrap values of clades are listed first, followed by Maximum Parsimony (MP) and Bayesian Posterior Probabilities, for clade frequencies exceeding 60%.

Phylogenetic trees inferred from a concatenated amino acid sequence dataset of 12 PCGs among selected enoplid nematodes, using the chromadorean nematode, *B. malayi* (NC_004298) and *A. suum* (HQ704901) as outgroups, revealed congruence with phylogenetics analyses of nucleotide sequence datasets with the 13 PCGs and the two rRNAs (*rrn*L and *rrn*S), and complete mt genomes. Thus, phylogenetics analyses of all 12 genes showed a general pattern with very strong support for clades near the terminals of the tree (Figure 4). Thus, clade 1, clade 2 and clade 3 for *Trichuris* spp. from humans and NHPs, and the monophyly of the genus *Trichuris,* are very strongly supported (100% ML BV, 100% MP BV and 100% BPP). There are no marked differences in support values between the 12 gene datasets analyzed using nucleotide versus amino acid sequences.

The phylogenetic analyses reflected the clear distinctiveness between *T. trichiura* and *Trichuris* sp. from humans and NHPs with respect to *T. suis* from suids, *T. ovis* and *T. discolor* from herbivorous and *T. muris* from rodents and grouping these members of *Trichuris* with *T. pseudospiralis* (order Trichinellida) with absolute support (100% ML BV, MP BV 100% and 100% BPP), but excluding the members of Dorylaimida and Mermithida (Figure 4).

## 4. Discussion

Previous studies have reported the hypothesis that a complex whipworm species exists in primates, suggesting that different *Trichuris* species infect primates and humans [3,19,23,38,39,40,41].

In the present study, we resolved the complete mt genome sequences of two haplotypes of *Trichuris* isolated from *M. sylvanus* and one complete mt genome of *Trichuris* from *P. papio*. The size of the complete mt genomes were within the range reported for the Trichuridae family (ranging from 13,904 bp (*T. discolor*) to 14,521 bp (*T. suis*)) [3,16,17,19,22]. The complete mtDNA is a circular molecule and encodes 37 genes—13 PCGs (*atp*6, *atp*8, *cox*1–3, *co*b, *nad*1–6 and *nad*4L), two rRNAs (*rrn*S and *rrn*L), and 22 for tRNAs.

The initiation codons (ATG, ATA and ATT) agreed with those reported in the pig-derived and human-derived *Trichuris*. [16,18,20], while *T. ovis* and *T. discolor* also employed TTG [3,16,19], and *T. skrjabini* sequence used only two start codons (ATG and ATA) [22]. Two termination codons (TAA and TAG) were used as stop codons in all the four species of Trichuridae family (*T. suis*, *T. trichiura*, *T. ovis* and *T. discolor*) except *T. skrjabini,* which possesses another stop codon, TGA [3,16,17,19,22]. The sequences of this study were in accordance with the most related species (primates derived *Trichuris*) [3,19].

The complete mt genomes of one of the haplotypes of *Trichuris* from macaques (TMF31) and that from baboon (TPM1) were genetically similar, with a difference in nucleotide and amino acid sequence of 0.25% and 0.41%, and having 14,091 and 14,098 bp, respectively, and both genomes being similar with that of *T. trichiura* from humans from Uganda, which was 14,079 bp in length, and with TTB1 (13,984 bp in length) from *Trichuris* sp. from *P. hamadryas* [19], with a sequence variation of 0.28–0.47%. In contrast, the complete mt genome of the other haplotype from macaques (TMM5) was 14,047 bp, like human *T. trichiura* from China that was 14,046 bp in length [3], showing a nucleotide difference of 15.9%. Moreover, the mt genome of *Trichuris* sp. from *P. anubis* (USA) was 14,009 bp [19], with a nucleotide difference to TMM5 of 15.8%. A substantial level of nucleotide difference was detected between TMF31 (Clade 3) with respect to *Trichuris* sp. included in clades 1 and 2 (15.8–18.8%). Comparison of the nucleotide and amino acid sequences between TMF31 and TMM5 was 18.7% and 14.5%, respectively. The sequence variation detected in the 13 PCGs between TMF31 and TMM5 was 20.3% (nucleotide sequence) and 34.4% (amino acid sequence). The percentage of dissimilarity observed between the different sequences corresponded to previously cited ranges within different nematode species. Thus, Hawash et al. [19] suggested the presence of two *Trichuris* species with a difference in nucleotide and amino acid sequences of around 18.8% and 14.6%, respectively, of *Trichuris* from Ugandan humans and Chinese humans. Furthermore, *T. suis* and *T. trichiura* presented an amino acid difference of 33.3–39.2% [19], *T. trichiura* from human and *Trichuris* sp. from leaf monkey was 29.4% [17], *T. ovis* and *T. discolor* ranged from 11.0–33.9% [16], 11.7% between *Wucheria bancrofti* and *B. malayi* [42], 10.3% between *Chabertia ovina* and *C. erschowi* [43], 4–18% between different *Trichinella* spp. [18] and 4.12% between *Ancylostoma duodenale* and *Ancylostoma caninum* [44]. Blouin [45] indicated that the difference in mt genome sequences between closely related species was normally 10–20%. Thus, the molecular evidence presented in the present manuscript supports the hypothesis that the haplotypes TMF31 and TMM5 of *Trichuris* from *M. sylvanus* display differences in amino acid and nucleotide sequences that are within the range of those previously reported by different authors to consider them as different species.

On the other hand, the complete mt genomes of *Trichuris* spp. from different hosts were evaluated to clarify the evolutionary relationships. Considering that complete mt genome sequences are maternally inherited and mutate at a rapid rate related to nuclear genes, theses markers could prove useful for evolutionary and phylogenetic analyses [46].

Phylogenetics trees inferred from both datasets (sequences dataset of 12 PCGs among enoplid nematodes and nucleotide sequence datasets with the 13 PCGs and the two rRNAs (*rrn*L and *rrn*S)) showed congruence with a high support for the differentiation between *Trichuris* spp. and the different clades within *Trichuris* populations parasitizing humans and NHPs (clade 1, clade 2 and clade 3). Furthermore, the monophyly of the genus *Trichuris* was strongly supported and grouping these species of *Trichuris* (traditionally named as Trichocephalida) with *T. pseuospirallis* (Trichinellida,) with the exclusion of the members of the Dorylaimida and Mermithida (Figure 4). Similar results were reported by Liu et al. [3,16] who characterized the complete mitochondrial genomes of several whipworms and compared them with other enoplid nematodes.

The *atp*8 gene is present in mitogenomes of the Order Trichinellida (*Trichuris* and *Trichinella* species), but it is usually absent in mitogenomes of members of the phylum Nematoda [3,13,15,16,17,18,19]. These authors reported the lack of *atp*8 in the mtDNA of Chromadorean nematodes (traditionally named as Class Secernentea) and it could be derived, within that lineage, after the divergence of Chromadorean nematodes from other groups. Furthermore, phylogenetic inferences confirm the clear relationships between Trichinellida and Mermithida correlating both groups with animal parasites of nematodes separated of Dorylaimida, corresponding to free-living nematodes and plant parasitic nematodes [47].

The phylogenetic study also supported the evolution of the different *Trichuris* species showing *T. trichiura* (clade 3), to be closer to *Trichuris* spp. (clade 1 and clade 2) parasitizing humans and NHPs. As reported by previous authors [19,23,38,39,41,48], *Trichuris* populations parasitizing humans and NHPs showed a species complex in primates. In addition, the high difference in nucleotide and amino acid sequences between *T. trichiura* (clade 3) and the minority population of *Trichuris* sp. from *M. sylvanus* (clade 2) and *Trichuris* sp. from humans and NHPs from China, Japan, and USA (clade 1) was confirmed in the phylogenetic trees with strong support for the different clades. Thus, based on our results, we suggest the existence of two cryptic species parasitizing *M. sylvanus*. *Trichuris* genus is a likely candidate to containing cryptic species as it has a wide geographical distribution and infects several host species [49]. As revealed by recent studies, there is more than one taxon capable of infecting humans and other primates, including individuals in captivity, suggesting that *T. trichiura* should be considered a complex species that includes different cryptic units [19]. In addition, and based on morphobiometric and molecular parameters, new species of *Trichuris* have been described in primates, such as *T. rhinopiptheroxella* [20], that was found in the golden snub-nosed monkey (*Rhinopithecus roxellana*), *Trichuris colobae* from *Colobus guereza kikuyensis* [50], and *Trichuris ursinus* from *Papio ursinus* [51]. As a result, it has been confirmed that *T. trichiura* and other *Trichuris* species are present in humans and NHPs.

This fact has two main epidemiological repercussions—(i) the zoonotic potential of *Trichuris* spp. of NHPs for humans. This is particularly important when humans and NHPs are living in proximity, as is becoming increasingly common with human encroachment into habitats where NHPs accessing gardens and farms in search of food and has significant implications for both human health and wildlife conservation [52]. This implies that in communities where access to NHPs is common, simple public health measures should be encouraged, including thorough handwashing with soap (particularly for children) and rising and cooking of vegetables, and (ii) the presence of different cryptic species might also be very important for implementation of appropriate control strategies. Different control strategies for *Opistorchirs viverrini* have been identified due to the existence of different cryptic species based on the different fecundity as measured by eggs/g/worm [53].

## 5. Conclusions

In the present study, based on complete mt genome analyses, the molecular data suggested two distinct species in whipworms isolated from *M. sylvanus*. The whipworms from *P. papio*, TPM1, and TMF31 from *M. sylvanus* are *T. trichiura* since the sequences were within the majority clade, with sequences from Ugandan humans and *P. hamadryas* (Europe)-derived *Trichuris*. Further, we suggested the existence of two cryptic species parasitizing *M. sylvanus*. Moreover, a major source of mitochondrial markers is given and may be used as the basis of subsequent epidemiological research, to clarify appropriate control measures and transmission routes.

## Figures and Tables

**Table 1:** Sequences analyzed in the first data set for phylogenetic analyses.

Species	Host Species/Geographical Origin	GenBank Accession Number
*Trichuris trichiura*	*Macaca sylvanus*/Spain	MW448470
*Trichuris* sp.	*Macaca sylvanus*/Spain	MW448471
*Trichuris trichiura*	*Papio papio*/Spain	MW448472
*Trichuris trichiura*	*Homo sapiens*/(unknown geographical location)	AP017704
*Trichuris trichiura*	*Homo sapiens*/China	NC_017750
*Trichuris trichiura*	*Homo sapiens*/China	GU385218
*Trichuris trichiura*	*Homo sapiens*/Uganda	KT449826
*Trichuris* sp.	*Papio anubis*/USA	KT449825
*Trichuris* sp.	*Papio hamadryas*/Denmark	KT449824
*Trichuris* sp.	*Trachypithecus francoisi*/China	KC461179
^1^ *Trichinella pseudospiralis*	*Coragypus atratus/USA*	KM357411

^1^ Used as an outgroup.

**Table 2:** Sequences analyzed in the second data set for phylogenetic analyses.

Species	Host Species/Geographical Origin	Order	GenBank Accession Number
*Trichuris trichiura*	*Macaca sylvanus*/Spain	Trichinellida	MW448470
*Trichuris* sp.	*Macaca sylvanus*/Spain	Trichinellida	MW448471
*Trichuris trichiura*	*Papio papio*/Spain	Trichinellida	MW448472
*Trichuris trichiura*	*Homo sapiens*/(unknown geographical location)	Trichinellida	AP017704
*Trichuris trichiura*	*Homo sapiens*/China	Trichinellida	NC_017750
*Trichuris trichiura*	*Homo sapiens*/China	Trichinellida	GU385218
*Trichuris trichiura*	*Homo sapiens*/Uganda	Trichinellida	KT449826
*Trichuris* sp.	*Papio anubis*/USA	Trichinellida	KT449825
*Trichuris* sp.	*Papio hamadryas*/Denmark	Trichinellida	KT449824
*Trichuris* sp.	*Trachypithecus francoisi*/China	Trichinellida	KC461179
*Trichuris rhinopiptheroxella*	*Rhinopithecus roxellana*/China	Trichinellida	MG189593
*Trichuris ovis*	*Addax nasomaculatus*/China	Trichinellida	NC_018597
*Trichuris discolor*	*Bos grunniens mutus*/China	Trichinellida	NC_018596
*Trichuris muris*	- /United Kingdom	Trichinellida	LC050561
*Trichuris suis*	*Sus scrofa*/Uganda	Trichinellida	KT449823
*Trichuris suis*	*Sus scrofa*/China	Trichinellida	GU070737
*Trichinella pseudospiralis*	*Coragypus atratus/USA*	Trichinellida	KM357411
*Xiphinema americanum*	*Plant ectoparasite*	Dorylaimida	NC_005928
*Hexamermis agrotis*	-	Mermithida	NC_008828
*Agamermis* sp.	-	Mermithida	NC_008231
*Romanomermis culicivorax*	-	Mermithida	NC_008640
*Romanomermis iyengari*	-	Mermithida	NC_008693
*Romanomermis nielseni*	-	Mermithida	NC_008692
*Strelkovimermis spiculatus*	-	Mermithida	NC_008047
*Thaumamermis cosgrovei*	-	Mermithida	NC_008046
^1^ *Brugia malayi*	-	Rhabditida	NC_004298
^1^ *Ascaris suum*	-	Rhabditida	HQ704901

^1^ Used as an outgroup.

**Table 3:** Mitochondrial genomes of *Trichuris* from *Macaca sylvanus* (TMF31, TMM5) and *Papio papio* (TPM1). Protein coding, transfer RNA (tRNA), and ribosomal RNA (rRNA) genes with lengths in nucleotides (nt) are given. The lengths are not identical, and differences are given in parentheses (TMM5/TPM1), likewise for the initiation and termination codons.

Genes	Positions			Lengths	Codons		Strand
	TMF31	TMM5	TPM1	nt	Initiation	Termination	
*cox*1	1–1545	1–1545	1–1545	1545	ATG	TAA (TAG)	+
*cox*2	1558–2232	1556–2230	1558–2232	675	ATG	TAG (TAA)	+
tRNA-leu (L_2_)	2255–2317	2252–2311	2255–2317	63 (60)			+
tRNA-glu (E)	2324–2384	2320–2377	2324–2384	61 (58)			+
*nad*1	2406–3305	2401–3300	2406–3305	900	ATA (ATG)	TAG (TAA)	+
Non-coding region (NCR-L)	3306–3435	3303–3442	3306–3430				
tRNA-lys (K)	3436–3501	3441–3502	3431–3496	66 (62)			+
*nad*2	3499–4395	3505–4401	3494–4390	897	ATA	TAA (TAG)	−
tRNA-met (M)	4396–4456	4402–4462	4391–4451	61			−
tRNA-phe (F)	4451–4507	4457–4513	4446–4502	57			−
*nad*5	4499–6055	4520–6067	4494–6050	1557 (1553)	ATA	TAG (TAA)	−
tRNA-his (H)	6049–6106	6065–6118	6044–6101	58 (54)			−
tRNA-arg (R)	6108–6171	6115–6181	6103–6166	64 (67)			−
*nad*4	6176–7396	6183–7394	6171–7391	1221 (1212)	ATG (ATA)	TAA	−
nad4L	7419–7631	7425–7673	7414–7626	213 (249)	ATA	TAA (TAG)	−
tRNA-thr (T)	7672–7729	7679–7736	7667–7724	58			+
tRNA-pro (P)	7729–7787	7736–7795	7724–7782	59 (60)			−
*nad*6	7780–8256	7788–8264	7775–8251	477	ATT	TAA (TAG)	+
*co*b	8263–9369	8272–9378	8258–9364	1107	ATG	TAG	+
tRNA-ser (S1)	9368–9420	9377–9426	9363–9415	53 (50)			+
*rrn*S	9413–10116	9419–10112	9408–10,111	704 (694)			+
tRNA-val (V)	10,118–10,174	10,114–10,170	10,113–10,169	57			+
*rrn*L	10,176–11,184	10,170–11,180	10,171–11,179	1009 (1011)			+
*atp*6	11,155–11,967	11,151–11,990	11,150–11,962	813 (840)	ATG	TAA	+
*cox*3	11,973–12,746	11,965–12,738	11,968–12,741	774	ATG	TAA (TAG)	+
tRNA-trp (W)	12,759–12,821	12,743–12,805	12,754–12,816	63			−
tRNA-gln (Q)	12,825–12,880	12,807–12,862	12,820–12,875	56			+
tRNA-Ile (I)	12,883–12,943	12,864–12,925	12,878–12,938	61 (62)			−
tRNA-gly (G)	12,957–13,013	12,934–12,989	12,952–13,008	57 (56)			−
tRNA-asp (D)	13,020–13,077	12,996–13,060	13,015–13,072	58 (65)			+
*atp*8	13,066–13,233	13,042–13,209	13,061–13,228	168	ATT (ATA)	TAG	+
*nad*3	13,243–13,584	13,219–13,560	13,238–13,579	342	ATT	TAA	+
Non-coding region NCR-S)	13,585–13,676	13,561–13,659	13,580–13,672				
tRNA-ser (S2)	13,677–13,726	13,660–13,709	13,673–13,723	50 (50/51)			+
tRNA-asn (N)	13,727–13,781	13,710–13,763	13,724–13,778	55 (54)			+
tRNA-leu (L1)	13,789–13,848	13,770–13,835	13,786–13,845	60 (66)			+
tRNA-ala (A)	13,860–13,917	13,842–13,897	13,857–13,914	58 (56)			+
tRNA-cys (C)	13,960–14,013	13,924–13,976	13,957–14,010	54 (53)			−
tRNA-tyr (Y)	14,014–14,074	13,977–14,038	14,011–14,071	61 (62)			−
**Total length**	14,091	14,047	14,089				

**Table 4:** Pairwise genetic and protein distances between the different complete mt genomes for different *Trichuris trichiura* and *Trichuris* sp. in different primates and human hosts at different countries. The nucleotide distances are given below the diagonal and the amino acid distances above the diagonal.

	TMF31	TPM1	TMM5	AP017704 *T. trichiura H. sapiens* (Unknown Geographical Location)	NC_017750 *T. trichiura H. sapiens* China	GU385218 *T. trichiura H. sapiens* China	KT2449826 *T. trichiura H. sapiens* Uganda	KT449825 *Trichuris* sp. TTB2 *P. anubis* USA	KT449824 *Trichuris* sp. *P. hamadryas* Denmark	KC461179 *Trichuris* sp. GHL *T. francoisi* China
TMF31		0.41	14.5	14.7	14.8	14.8	0.6	14.6	0.49	26.9
TPM1	0.25		14.6	14.8	14.9	14.9	0.7	14.6	0.34	26.8
TMM5	18.7	18.7		10.9	11.1	11.1	14.5	10.3	14.6	28.2
AP017704 *T. trichiura H. sapiens* (unknown geographical location)	18.7	18.7	15.7		4.68	4.68	14.7	3.47	14.9	28.3
NC_017750 *T. trichiura H. sapiens* China	18.6	18.6	15.9	6.51		0	14.8	4.66	14.9	27.8
GU385218 *T. trichiura H. sapiens* China	18.6	18.6	15.9	6.51	0		14.8	4.66	14.9	27.8
KT2449826 *T. trichiura H. sapiens* Uganda	0.4	0.47	18.8	18.7	18.6	18.6		14.5	0.78	26.8
KT449825 *Trichuris* sp. TTB2 *P. anubis* USA	18.8	18.8	15.8	4.7	6.48	6.48	18.8		14.7	28.2
KT449824 *Trichuris* sp. *P. hamadryas* Denmark	0.34	0.28	18.8	18.7	18.6	18.6	0.55	18.8		26.8
KC461179 *Trichuris* sp. GHL *T. francoisi* China	27.1	27.1	28.4	28.3	28	28	27.1	28.6	27.2	

## Data Availability

The data presented in this study are openly available in GenBank database (GenBank accession nos. MW448470-2).

## Supplementary Materials

The following are available online at https://www.mdpi.com/2075-1729/11/2/126/s1, Table S1: PCR mix, primers and conditions used for each molecular marker sequenced in the present study, Table S2: Pairwise nucleotide and amino acid distances for the different 13 PCGs and RNAs (*rrn*S and *rrn*L) genes for *Trichuris* species from different primates and human hosts. Nucleotide genetics distances are given below the diagonal and amino acid genetic distances above the diagonal, Table S3: Nucleotide composition (%) of the mt genomes studied.
