# Supplementary material for: Complete Mitochondrial Genome of Trichuris trichiura from Macaca sylvanus and Papio papio
Source: Life (Basel). 2021 Feb 6;11(2):126. doi: 10.3390/life11020126 (PMC7915941; doi:10.3390/life11020126)
Supplement: Supplementary file 1 [file life-11-00126-s001.zip › Table S1.docx]

| PCR Mix | | |
| --- | --- | --- |
| Forward Primer (10 µM) | 2.5 µl |  |
| Reverse Primer (10 µM) | 2.5 µl |  |
| Q5^®^ High-Fidelity 2X Master Mix | 25 µl |  |
| Template DNA | 5 µl |  |
| Nuclease free water to | 50 µl |  |
| PCR Primers | | |
|  | 1º fragment | 2º fragment |
| Forward Primer | MS1F: 5’-ACARCCCATYCTAGACGGTA-3’ | TTB1rrnLF (Hawash et al., 2015) |
| Reverse Primer | MS1R: 5’-AATTCCCAGGGTCTTCTCGT-3’ | TTB1nad1R (Hawash et al., 2015) |
| PCR Conditions | | |
|  | 1º fragment | 2º fragment |
| Initial Denaturing | 92° C/4 min | 92° C/4 min |
| Number of cycles | 35 | 35 |
| Denaturing | 92° C/20 s | 92° C/20 s |
| Annealing | 59° C/30 s | 50° C/30 s |
| Primer extension | 67° C/7 min | 67° C/7 min |
| Final extension | 67° C/10 min | 67° C/10 min |

**Table S1.** PCR mix, primers and conditions used for the complete mitochondrial genomes sequenced in the present study.
