# Supplementary material for: Complete Mitochondrial Genome of Trichuris trichiura from Macaca sylvanus and Papio papio
Source: Life (Basel). 2021 Feb 6;11(2):126. doi: 10.3390/life11020126 (PMC7915941; doi:10.3390/life11020126)
Supplement: Supplementary file 1 [file life-11-00126-s001.zip › Table S3.docx]

**Table S3.** Nucleotide composition (%) of the mt genomes studied.

| ***Trichuris* genomes** | **T** | **C** | **A** | **G** | **A+T** |
| --- | --- | --- | --- | --- | --- |
| TMF31 | 35.1 | 16.4 | 34.3 | 14.3 | 69.4 |
| TMM5 | 35.0 | 16.3 | 33.0 | 15.7 | 68 |
| TPM1 | 35.0 | 16.4 | 34.2 | 14.3 | 69.3 |
